# Margin Accentuation Irreversible Electroporation in Stage III Pancreatic Cancer: A Systematic Review

**DOI:** 10.3390/cancers13133212

**Published:** 2021-06-27

**Authors:** Bathiya Ratnayake, Dhya Al-Leswas, Ghazaleh Mohammadi-Zaniani, Peter Littler, Gourab Sen, Derek Manas, Sanjay Pandanaboyana

**Affiliations:** 1 Department of Surgery, Faculty of Medical and Health Sciences, University of Auckland, Auckland 1023, New Zealand; Crat791@aucklanduni.ac.nz; 2 Hepato-Pancreato-Biliary and Transplant Unit, Freeman Hospital, Newcastle upon Tyne NE7 7DN, UK; dhya@doctors.org.uk (D.A.-L.); g.zaniani@nhs.net (G.M.-Z.); gourab.sen@nhs.net (G.S.); derek.manas@nhs.uk (D.M.); 3 Department of Interventional Radiology, Freeman Hospital, Newcastle upon Tyne NE7 7DN, UK; peter.littler@nhs.net; 4 Population Health Sciences Institute, Newcastle University, Newcastle upon Tyne NE2 4AX, UK

**Keywords:** irreversible electroporation, margin accentuation, pancreatic cancer, pancreatic surgery

## Abstract

**Simple Summary:**

This literature review shows preliminary evidence to suggest that electroporation, the use of electricity to cause the death of cells around the tumour, may be associated with an improved survival and complete resection rates following pancreatic surgery for higher stage pancreatic cancer. However, one in five patients have a complication from the procedure that alters their normal course in hospital. Moreover, the number of patients who underwent this technique is small and further data is needed to support the preliminary evidence. The results therefore should be interpreted with caution.

**Abstract:**

The present systematic review aimed to summarise the available evidence on indications and oncological outcomes after MA IRE for stage III pancreatic cancer (PC). A literature search was performed in the Pubmed, MEDLINE, EMBASE, SCOPUS databases using the PRISMA framework to identify all MA IRE studies. Nine studies with 235 locally advanced (LA) (82%, 192/235) or Borderline resectable (BR) PC (18%, 43/235) patients undergoing MA IRE pancreatic resection were included. Patients were mostly male (56%) with a weighted-mean age of 61 years (95% CI: 58–64). Pancreatoduodenectomy was performed in 51% (120/235) and distal pancreatectomy in 49% (115/235). R0 resection rate was 73% (77/105). Clavien Dindo grade 3–5 postoperative complications occurred in 19% (36/187). Follow-up intervals ranged from 3 to 29 months. Local and systematic recurrences were noted in 8 and 43 patients, respectively. The weighted-mean progression free survival was 11 months (95% CI: 7–15). The weighted-mean overall survival was 22 months (95% CI 20–23 months) and 8 months (95% CI 1–32 months) for MA IRE and IRE alone, respectively. Early non-randomised data suggest MA IRE during pancreatic surgery for stage III pancreatic cancer may result in increased R0 resection rates and improved OS with acceptable postoperative morbidity. Further, larger studies are warranted to corroborate this evidence.

## 1. Introduction

Pancreatic cancer is one of the most aggressive malignancies and the seventh leading cause of cancer-related death worldwide [1]. Surgical resection remains the only effective potential curative therapy but only 10–20% are amenable for resection at the time of diagnosis [2]. This is due to the fact that around 85% of patients present with locally advanced disease (stage three) or have metastases (stage four) [3].

Stage three pancreatic cancer is defined as a cancer that involves major vascular structures and is further subcategorised based on the extent of the vascular involvement into borderline resectable (BRPC) and locally advanced unresectable pancreatic cancer (LAPC) [4]. BRPC with venous involvement is often considered resectable, on the contrary BRPC with arterial involvement is often considered for neoadjuvant chemotherapy. Patients with LAPC are considered for neoadjuvant therapy (NAT) first to downstage the tumours for potential curative resection in the future. The introduction of FOLFIRINOX NAT has led to resectability rates up to 10–35% for LAPC with recent nonrandomised cohort studies reporting a survival of 30–34 months from diagnosis for patients undergoing resection after FOLFIRINOX [5,6]. Determining radiological resectability in patients after NAT for LAPC is often difficult [7] and surgery is considered if there is stable disease or no progression on the RECIST criteria with a falling CA19-9 [8]. In patients who undergo resection, R1 rates range between 14% and 100% [9]. It is well documented that NAT causes extensive pathological changes in the pancreatic gland, resulting in a higher extent of fibrosis and pancreatic atrophy. This results in difficult pathological assessment leading to an overestimation of R0-rates since sparse tumour cells may skip the resection margin [10]. Local ablative techniques such as irreversible electroporation (IRE), radiofrequency ablation (RFA), and stereotactic body radiation therapy (SBRT) are being increasingly used in patients with LAPC as an alternative to surgical resection to avoid futile margin positive resections and to prolong survival and minimise surgical morbidity.

Contrary to other ablation methods, IRE generates an electric field through two or more electrodes that are inserted around the tumour with multiple cycles of short, high-voltage electrical pulses generated across the ablation zone. This alters the potential of the tumour cells transmembrane creating defects in the cell membrane leading to increase membrane permeability and loss of cell homeostasis [11]. This immune mediated cell death allows for cellular clearance of this debris and causes minimal distortion to the nearby tissues and vital structures such as SMA and portal vein [12,13]. IRE (percutaneous, laparoscopically or open) used as an ablative procedure for LAPC, has been shown to be safe and may improve overall survival and progression free survival [14]. More recently, IRE has expanded to “margin accentuation IRE” (MA IRE). MA IRE is typically used in patients with LAPC or BRPC intraoperatively during pancreatic resection to achieve a true R0 resection. The aim of this review is to analyse the outcomes following MA IRE pancreatic resection.

## 2. Materials and Methods

This systematic review was conducted following the recommendations of the Preferred Reporting Items for Systematic Reviews and Meta-Analyses (PRISMA) guidelines [15]. The protocol of this review was registered on the Prospero database (ID: CRD42020221643).

### 2.1. Literature Search

A systematic search was developed and the PubMed, EMBASE, SCOPUS and Cochrane library were queried using the following search terms combined with their respective Boolean operators; the combined results of “irreversible electroporation” OR “IRE” AND the combined results of “pancreas” OR “pancreatic resection” OR “Whipple’s procedure” OR “Pylorus-preserving pancreaticoduodenectomy (PPPD)” in human studies published in English from 1 January 2005 until 28 January 2021. Articles that reported on MA IRE on patients with LAPC or BRPC were identified and the reference lists were further evaluated to identify additional studies missed by the primary search strategy.

### 2.2. Inclusion and Exclusion Criteria

Original articles were included if they reported on outcomes following MA IRE in patients with LAPC or BRPC with subsequent pancreatic resection. Inclusion was limited to English articles and included case reports, cohort studies, and randomised controlled trials (RCTs). Criteria for exclusion included non-English studies, reviews, letters, abstracts, palliative IRE treatment alone, studies in benign tumours or animals, and laboratory studies.

### 2.3. Data Selection and Extraction

Two authors screened through the title, abstracts and full texts of all articles identified in the primary search strategy and then subsequently performed the data extraction (BR and DA). Enduring conflicts were resolved following review by a third author (SP). Extracted perioperative and operative variables included demographic data (age and sex), tumour histology, stage (LAPC or BRPC), vascular involvement, chemotherapy in the neoadjuvant and adjuvant setting, associated chemoradiotherapy, postoperative morbidity, R0 resection, follow-up interval, progression free survival, and overall survival. The primary endpoint was overall survival. Secondary endpoints included overall morbidity, recurrence rates (local and systemic) and R0 resection rate.

### 2.4. Definitions

LAPC was defined as greater than 180 involvement of the celiac artery, SMA or both without metastatic disease [16]. BRPC was defined as tumour involvement of less than 180 of the total circumference of the celiac axis or SMA, short-segment hepatic artery involvement, or occlusion of the superior mesenteric vein (SMV), portal vein (PV), or SMV–PV confluence in a short segment with the potential for resection and reconstruction of the vessel [17]. Complications were reported as per the Clavien Dindo classification [18]. Overall survival was defined as the interval from diagnosis or, where available, from IRE administration [19]. R0 resection was defined as microscopically negative margins following resection, R1 resection having microscopically positive margins and R2 showing significant residual disease [20]. Peritoneal recurrence was defined as suspicious omental or peritoneal lymph nodes or the presence of new ascites [17].

### 2.5. Statistical Methodology and Risk of Bias Assessment

An inverse variance method using the R studio package “MetaAnalyser” [21] for the calculation of weighted means and respective 95% confidence intervals (CI) in R project (R Foundation for Statistical Computing, Austria 2014). Survival data was extracted with webplotdigitizer [22] and mean and standard deviation estimates from the extracted median and ranges, or confidence intervals were obtained previously validated methodology [23]. The risk of bias and study quality assessment was performed through use of the methodological index for non-randomised cohort studies (MINORS) grading criteria for non-randomised studies [24].

## 3. Results

**Figure 1:**
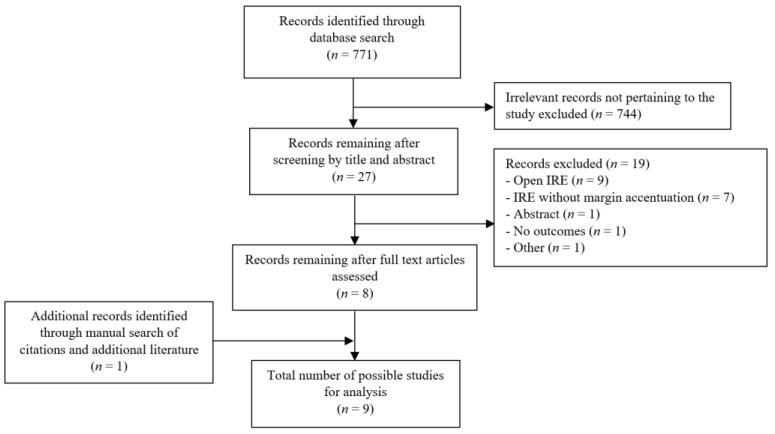
PRISMA flow chart of literature search strategy.

In total, 771 articles were retrieved from the database search. Among them, nine studies [13,17,19,25,26,27,28,29,30] fulfilled the inclusion criteria and were included in the quantitative analysis (Figure 1). The included studies were largely observational (*n* = 5) [13,17,25,29,30] and comparative (*n* = 3) [19,26,28] cohort studies, in addition to a single case report (*n* = 1) [27]. Articles were published in United States (*n* = 7) [13,17,19,25,26,28,30] and Europe (*n* = 2) [27,29]. A total of 593 patients with stage 3 LAPC or BRPC were subjects for interventions in these studies, among them, 235 patients had margin accentuation IRE (MA IRE) pancreatic resection (Table 1). Patients were predominantly male (56%, 75/134) in five studies [17,25,27,28,29] reporting gender distribution, with a weighted mean age for the entire MA IRE cohort of 61 years (95% CI: 58–64 years) [17,19,25,28]. LAPC comprised the significant majority of the cohort (82%, 192/235), however, 43 BRPC (18%) patients were present in three studies [17,27,29] (Table 2). LAPC was defined as per the 7th edition of the American Joint Committee on Cancer (AJCC) staging system [16,31] consistently in 6/7 studies [13,17,19,25,26,30] and BRPC was consistently defined in all three studies [17,27,29]. One study did not report the definition of LAPC [28].

All patients received NAT prior to MA IRE, however, only five studies [17,19,25,27,28] reported specific regimens for the MA IRE cohort. Gemcitabine- (55% 98/179) and Folfirinox-based (35% 62/179) were the most common therapies utilised. The duration of NAT was reported in two studies: 12 cycles for all patients [27] and a median of 6 cycles (range 6–8 cycles) [25], respectively. Similarly, the median time of IRE procedure from diagnosis was reported in two studies: 6 months (range 4–13) [17] and 5.2 months (range 3–18) [19], respectively. The decision to perform MA IRE pancreatic resection or IRE alone was at the surgeon’s discretion based on intraoperative assessment, patient comorbidities, previous therapy, and patient choice. In general, patients with suspected R1 resection were candidates for MA IRE pancreatic resection and patients that are likely to achieve R2 resection were candidates for IRE alone procedure (open or percutaneous).

### 3.1. Tumour Characteristics and Pancreatic Resections

Head or uncinate process tumours were present in 50% (118/235) of patients and the remainder of tumours were located in the neck or body (50%, 117/235). Vascular involvement was present in 97% (228/235) of patients. Pancreatoduodenectomy (classical pancreaticoduodenectomy or pylorus preserving pancreaticoduodenectomy) was performed in 51% (120/235) of patients and a subtotal or distal pancreatoduodenectomy was performed in 49% (115/235) (Table 2). Arterial resection was performed in 46% (66/142) and venous resection in 44% (63/142) in five studies [17,19,26,27,28] reporting specific vascular resections performed at the time of pancreatectomy.

### 3.2. Survival and Recurrence

The follow-up intervals ranged from 3 to 29 months in all included studies with the exception of one study [26], however, a weighted analysis was not performed given the variability in reporting. Overall recurrence rate was 33% (63/192) in seven studies [13,17,19,25,27,29,30] (Table 3). Five percent (3/63) of overall recurrences were observed within the first 12 months of follow-up, the remainder were observed following the first 12 months. Among those with a recurrence (*n* = 63), local recurrence was observed in eight patients (13%), peritoneal recurrence in 13 (21%) and distant metastases in 43 (68%) patients. Two studies [19,28] compared survival outcomes between MA IRE and IRE alone, one failed to reach median overall survival during their follow-up interval of 8.69 months [28]. The weighted mean progression free survival was 11 months (95% CI: 7–15 months) in two studies [17,25]. The weighted mean overall survival in the MA IRE cohort was 22 months (95% CI 20–23 months) in three studies [17,19,25] and the weighted mean overall survival in the IRE alone cohort was 8 months (95% CI 1–32 months) in two studies [13,28] (Table 3).

### 3.3. Pathological Outcomes, Complications and Length of Stay

R0 resection rate was 73% (77/105) in three studies [17,25,27]. Postoperative complications were reported for MA IRE in six studies [13,17,19,27,28,29] and occurred in 29% (55/187) of patients. Clavien Dindo grade 3–5 postoperative complications occurred in 19% (36/187) of patients in six studies [13,17,19,27,28,29] with a postoperative mortality observed in 2% (3/187). Six patients developed portal vein/superior mesenteric vein thrombus in four studies [17,27,28,29] (8%, 6/78). Four studies [17,19,25,28] reported the length of hospital stay in the MA IRE groups with a weighted mean of 13 days (95% CI: 9–17 days) (Table 3).

### 3.4. Heterogeneity and Risk of Bias

Overall, the non-randomised observational (median 13/16, range 7–13) cohort studies scored moderately in the MINORS criteria, however, the comparative cohorts performed relatively poorly (median 9/26, range 7–11). The lack of power calculations, adequate controls and contemporary study populations were consistently poorly performing domains within these included comparative cohorts [19,26,28] (Table S1).

## 4. Discussion

The present systematic review and metanalysis summarised the available evidence for margin accentuation IRE during pancreatic resection in a cohort of 235 patients with stage III pancreatic cancer undergoing NAT. Margin accentuation IRE resulted in a progression free survival of 11 months and an overall survival of 22 months. Although R0 resection was achieved in 73%, a third of patients developed recurrence during follow up with systematic recurrences more frequent than local recurrence. The postoperative morbidity was acceptable (30%), albeit with a high postoperative mortality (8%).

Despite advances in chemotherapy regimens and radiotherapy, inoperable LAPC has a poor median overall survival of 6–11.5 months in the majority of prospective clinical trials [32]. In these patients, after induction chemotherapy, ablative techniques such as radiofrequency ablation (RFA), microwave ablation (WMA), high intensity focused ultrasound (HIFU), cryoablation and irreversible electroporation (IRE) may provide symptomatic relief, a survival benefit, and downsize tumours. However, the majority of these ablative techniques utilise thermal energy with a potential to damage PV-SMV, SMA and bile ducts resulting venous and arterial thrombosis, fistulae or bile leaks. On the contrary, IRE destroys cancerous cells by delivering short electric pulses through electrodes inserted directly into the targeted tumours. Prior studies [33,34] have shown that IRE induces cell death in targeted cancerous cells while maintaining the integrity of the stromal elements of the tissue such that in locally advanced pancreatic cancer SMV, SMA/Coeliac axis are not thrombosed or strictured when IRE is appropriately performed. Martin et al. [26] first reported the oncological benefits of IRE in a series of 54 patients with LAPC. An improvement in the local progression-free survival (14 vs. 6 months, *p* = 0.01), distant progression-free survival (15 vs. 9 months, *p* = 0.02), and overall survival (20 vs.13 months, *p* = 0.03) was observed in patients with LAPC treated with IRE and chemotherapy versus chemotherapy alone [26]. Several studies including systematic reviews confirmed the benefits of IRE in patients in inoperable LAPC with a median OS ranging from 10 to 27 months [35]. The majority of studies, however, included patients undergoing IRE alone without surgical resection of the primary tumour called ‘in site IRE’ as opposed to patients where IRE was used as an adjunct to surgery intraoperatively aiming to achieve a higher percentage of R0 resection along SMV and SMA margin termed as ‘margin accentuation IRE’.

The weighted mean progression free survival and overall survival for MA IRE in the present review were 11 and 22 months, respectively, when compared to overall survival of 8 months with in site IRE. Martin et al. in a series of 200 patients with non-progressive LAPC who were treated with IRE alone (*n* = 150) and MA IRE (*n* = 50) showed a median overall survival of 28.3 months for MA IRE group and 23.2 months for the in situ IRE group [19]. However, these results were not reproduced by other centres. Kruger et al. [28] in a series of 50 patients undergoing 53 IRE procedures showed a median overall survival of 7.71 months for in site IRE and the median was not reached in the margin accentuation group. The R0 resection rates after MA IRE from the present review were 73%. Although initial series reporting R0 after FOLFORINOX based chemotherapy showed promise, more recent data from high volume centres after standardisation of pathological reporting have shown R0 resection rates between 30% and 50% [36,37]. Margin status after NAT has impact on survival [36] and it remains to be seen if the improved margin status after MA IRE will result in prolonged survival. The predominant site of recurrence during follow up in the present series after MA IRE was distant recurrence (liver). Although NAT [38] and MA IRE may result in lower rates of loco-regional recurrence, the distance recurrence patterns appear to be unchanged with recurrence in the liver still common. The variable follow up intervals in the present review did not allow calculation of time to recurrence in the MA IRE group.

There was also paucity of data on adjuvant chemotherapy or immunotherapy after MA IRE in the present series. Recently, Scheffer et al. showed that IRE alleviates the immunosuppression induced by LAPC by reducing systemic Treg populations and activating PD-1+ T cells [39]. The Treg rates drop by 24h and remain significantly decreased until at least two weeks post-IRE; after three months Treg frequencies appear to be recovering. This points to a transient but actionable therapeutic window in which tumour-related immune suppression appears to lift. These results suggest that IRE may create a temporary window for the successful application of immunotherapy in LAPC, in effect serving as a means of in vivo vaccination. In animal models, the combination of IRE with a checkpoint inhibition and TLR7 agonist, not only improved the local effects of IRE but also generated therapeutic abscopal effects against small secondary tumours, modelling the potential eradication of distant micrometastatic disease [40]. These findings have potential implications for increased used of in site IRE first to allow check point inhibitor use to downregulate the tumours and subsequent surgery with survival benefit. Similarly, the use of reversible electrochemotherapy has also been proposed as an additional non-thermal ablation technique to improve rates of local disease control and overall survival in LAPC [41,42,43]. Here, permeabilising electrical pulses coupled with a bleomycin infusion provides cytotoxic therapy delivery to cancer cells but limits systemic side effects [43].

There were several limitations in the methodology and available datasets. Given the emerging nature of MA IRE, it comes as no surprise that the review is composed mainly from non-randomised, retrospective, low powered, and observational datasets thereby limiting the comparability of included cohorts. Power limitations further restricted the ability to investigate the relative impact of MA IRE in LAPC vs. BRPC and were therefore combined in the outcome assessments. Furthermore, there remains no standardised indication for MA IRE in pancreatic cancer surgery and so intercohort variances in tumour stage and management do exist. Outcomes are further confounded by the lack of sufficient reporting on adjuvant therapy regimens following resection. Despite these limitations, this is the first review to summarise the currently available evidence of MA IRE.

## 5. Conclusions

In conclusion, there is early non-randomised evidence to suggest margin accentuation can improve R0 resection rates and OS in patients with LAPC with acceptable postoperative morbidity. Further larger studies are warranted to confirm the benefits of MA IRE in patients with LAPC undergoing pancreatic resection.

## Figures and Tables

**Table 1:** Study characteristics of all articles reporting on margin accentuation irreversible electroporation for locally advanced pancreatic cancer.

Author	Publication Year	Type of Study	Location of Publication	Total Cohort	MA IRE Cohort
Kluger et al. [25]	2018	Observational Cohort	USA	56	56
Papoulas et al. [27]	2018	Case Report	United Kingdom	1	1
Marsanic et al. [29]	2017	Observational Cohort	Italy	7	5
Kluger et al. [28]	2016	Comparative Cohort	USA	50	24
Martin et al. [19]	2015	Comparative Cohort	USA	200	50
Dunki-Jacobs et al. [30]	2014	Observational Cohort	USA	65	24
Kwon et al. [17]	2014	Observational Cohort	USA	48	48
Martin et al. [26]	2013	Comparative Cohort	USA	139	19
Martin et al. [13]	2012	Observational Cohort	USA	27	8

MA IRE—margin accentuation irreversible electroporation; USA—United States of America.

**Table 2:** Tumour and treatment characteristics of all included studies.

Author	Publication Year	LAPC/BRPC	Neoadjuvant	Vascular Involvement	PD/DP	Arterial/Venous Resection
Kluger et al. [25]	2018	56/0	56	49	34/22	-
Papoulas et al. [27]	2018	0/1	1	1	1/0	0/1
Marsanic et al. [29]	2017	0/5	5	5	5/0	-
Kluger et al. [28]	2016	24/0	22	24	15/9	0/12
Martin et al. [19]	2015	50/0	8	50	13/37	37/25
Dunki-Jacobs et al. [30]	2014	24/0	24	24	8/16	-
Kwon et al. [17]	2014	11/37	18	48	31/17	10/25
Martin et al. [26]	2013	19/0	19	19	9/10	19/0
Martin et al. [13]	2012	8/0	8	8	4/4	-

LAPC—locally advanced pancreatic cancer; BRPC—borderline resectable pancreatic cancer; PD—pancreaticoduodenectomy; DP—distal pancreatectomy; - not reported.

**Table 3:** Outcomes reported in all included studies.

Author	Publication Year	MA IRE Cohort	R0 Resection	LOS (Days) *	PFS (Months) ^¥^	Overall Recurrence	Overall Survival MA IRE (Months) ^¥^	Overall Survival No MA IRE Cohort (Months) ^¥^
Kluger et al. [25]	2018	56	45	7 (5–11)	8.5 (6–15)	26	18.5 (12–32)	-
Papoulas et al. [27]	2018	1	1	-	-	0	-	-
Marsanic et al. [29]	2017	5	-	-	-	0	-	-
Kluger et al. [28]	2016	24	-	8 (3–40)	-	-	-	7.7 (6–12)
Martin et al. [19]	2015	50	-	7 (4–26)	-	6	28.3 (9–85) *	23.2 (5–76) *
Dunki-Jacobs et al. [30]	2014	24	-	6 (5–58)	-	3	-	-
Kwon et al. [17]	2014	48	31	9 (4–58)	10.7 (3–30)	28	22.4 (18–25)	-
Martin et al. [26]	2013	19	-	-	-	-	-	-
Martin et al. [13]	2012	8	-	-	-	0	-	-

MA IRE—margin accentuation irreversible electroporation; LOS—length of stay; PFS—progression free survival; * originally extracted data presented with median (range) values; ^¥^ originally extracted data presented with medians (95% confidence intervals); - not reported.

## Supplementary Materials

The following are available online at https://www.mdpi.com/article/10.3390/cancers13133212/s1, Table S1. Methodological items for non-randomised studies criteria (MINORS) scores among all domains for all included studies.

## References

[1] Rawla P., Sunkara T., Gaduputi V. (2019). Epidemiology of Pancreatic Cancer: Global Trends, Etiology and Risk Factors. World J. Oncol..

[2] Bilimora K.Y., Bentrem D.J., Ko C.Y., Steewart A.K., Winchester D.P., Talamonti M.S. (2007). National Failure to Operate on Early Stage Pancreatic Cancer. Ann. Surg..

[3] Perysinakis I., Avlonitis S., Georgiadou D., Tsipras H., Margaris I. (2015). Five-year actual survival after pancreatoduodenectomy for pancreatic head cancer. ANZ J. Surg..

[4] Tempero M.A., Malafa M.P., Behrman S.W., Benson A.B., Casper E.S., Chiorean E.G., Chung V., Cohen S.J., Czito B., Engebretson A. (2014). Pancreatic adenocarcinoma, version 2.2014: Featured updates to the NCCN guidelines. J. Natl. Compr. Cancer Netw..

[5] Rombouts S.J., Mungroop T.H., Heilmann M.N., van Laarhoven H.W., Busch O.R., Molenaar I.Q., Besselink M.G., Wilmink J.W. (2016). FOLFIRINOX in Locally Advanced and Metastatic Pancreatic Cancer: A Single Centre Cohort Study. J. Cancer.

[6] Suker M., Beumer B.R., Sadot E., Marthey L., Faris J.E., Mellon E.A., El-Rayes B.F., Wang-Gillam A., Lacy J., Hosein P.J. (2016). FOLFIRINOX for locally advanced pancreatic cancer: A systematic review and patient-level meta-analysis. Lancet Oncol..

[7] Barreto S.G., Loveday B., Windsor J.A., Pandanaboyana S. (2019). Detecting tumour response and predicting resectability after neoadjuvant therapy for borderline resectable and locally advanced pancreatic cancer. ANZ J. Surg..

[8] Eisenhauer E.A., Therasse P., Bogaerts J., Schwartz L.H., Sargent D., Ford R., Dancey J., Arbuck S., Gwyther S., Mooney M. (2008). New response evaluation criteria in solid tumours: Revised RECIST guideline (version 1.1). Eur. J. Cancer.

[9] Hank T., Strobel O. (2019). Conversion Surgery for Advanced Pancreatic Cancer. J. Clin. Med..

[10] Verbeke C., Löhr M., Severin Karlsson J., Del Chiaro M. (2014). Pathology reporting of pancreatic cancer following neoadjuvant therapy: Challenges and uncertainties. Cancer Treat. Rev..

[11] Lee E.W., Thai S., Kee S.T. (2010). Irreversible Electroporation: A Novel Image-Guided Cancer Therapy. Gut Liver.

[12] Cannon R., Ellis S., Hayes D., Narayanan G., Martin R.C.G. (2013). Safety and early efficacy of irreversible electroporation for hepatic tumors in proximity to vital structures. J. Surg. Oncol..

[13] Martin R.C.G., McFarland K., Ellis S., Velanovich V. (2012). Irreversible Electroporation Therapy in the Management of Locally Advanced Pancreatic Adenocarcinoma. J. Am. Coll. Surg..

[14] Ansari D., Kristoffersson S., Andersson R., Bergenfeldt M. (2017). The role of irreversible electroporation (IRE) for locally advanced pancreatic cancer: A systematic review of safety and efficacy. Scand. J. Gastroenterol..

[15] Moher D., Liberati A., Tetzlaff J., Altman D.G., Group P. (2009). Preferred reporting items for systematic reviews and meta-analyses: The PRISMA statement. PLoS Med..

[16] Callery M.P., Chang K.J., Fishman E.K., Talamonti M.S., William Traverso L., Linehan D.C. (2009). Pretreatment Assessment of Resectable and Borderline Resectable Pancreatic Cancer: Expert Consensus Statement. Ann. Surg. Oncol..

[17] Kwon D., McFarland K., Velanovich V., Martin R. (2014). Borderline and locally advanced pancreatic adenocarcinoma margin accentuation with intraoperative irreversible electroporation. Surgery.

[18] Clavien P.A., Barkun J., Graf R., Vonlanthen R., Padbury R., Cameron K.L., De Oliveira M.L., Vauthey J.N., Dindo D., Schulick R.D. (2009). The Clavien-Dindo Classification of Surgical Complications: Five-Year Experience. Ann. Surg..

[19] Martin R.C., Kwon D., Chalikonda S., Sellers M., Kotz E., Scoggins C., McMasters K.M., Watkins K. (2015). Treatment of 200 Locally Advanced (Stage III) Pancreatic Adenocarcinoma Patients with Irreversible Electroporation: Safety and Efficacy. Ann. Surg..

[20] Hermanek P., Wittekind C. (1994). Residual tumor (R) classification and prognosis. Semin. Surg. Oncol..

[21] Bowden J., Jackson C. (2016). MetaAnalyser: An Interactive Visualisation of Meta-Analysis as a Physical Weighing Machine. https://cran.r-project.org/web/packages/MetaAnalyser/index.html.

[22] Ankit R. (2017). WebPlotDigitizer 4.0. https://automeris.io/WebPlotDigitizer.

[23] Wan X., Wang W., Liu J., Tong T. (2014). Estimating the sample mean and standard deviation from the sample size, median, range and/or interquartile range. BMC Med. Res. Methodol..

[24] Slim K., Nini E., Forestier D., Kwiatkowski F., Panis Y., Chipponi J. (2003). Methodological index for non-randomized studies (MINORS): Development and validation of a new instrument. ANZ J. Surg..

[25] Kluger M.D., Rashid M.F., Rosario V.L., Schrope B.A., Steinman J.A., Hecht E.M., Chabot J.A. (2018). Resection of Locally Advanced Pancreatic Cancer without Regression of Arterial Encasement After Modern-Era Neoadjuvant. Ther. J. Gastrointest. Surg..

[26] Martin R.C.G., McFarland K., Ellis S., Velanovich V. (2013). Irreversible Electroporation in Locally Advanced Pancreatic Cancer: Potential Improved Overall Survival. Ann. Surg. Oncol..

[27] Papoulas M., Abdul-Hamid S., Peddu P., Cotoi C., Heaton N., Menon K. (2018). Irreversible electroporation in borderline resectable pancreatic adenocarcinoma for margin accentuation. J. Surg. Case Rep..

[28] Kluger M.D., Epelboym I., Schrope B.A., Mahendraraj K., Hecht E.M., Susman J., Weintraub J.L., Chabot J.A. (2016). Single-Institution Experience with Irreversible Electroporation for T4 Pancreatic Cancer: First 50 Patients. Ann. Surg. Oncol..

[29] Marsanic P., Mellano A., Sottile A., De Simone M. (2017). Irreversible electroporation as treatment of locally advanced and as margin accentuation in borderline resectable pancreatic adenocarcinoma. Med. Biol. Eng. Comput..

[30] Dunki-Jacobs E.M., Philips P., Martin R.C. (2014). Evaluation of Resistance as a Measure of Successful Tumor Ablation During Irreversible Electroporation of the Pancreas. J. Am. Coll. Surg..

[31] Varadhachary G., Tamm E., Abbruzzese J., Xiong H., Crane C., Wang H., Lee J.E., Pisters P.W., Evans D.B., Wolff R.A. (2006). Borderline Resectable Pancreatic Cancer: Definitions, Management, and Role of Preoperative Therapy. Ann. Surg. Oncol..

[32] Paiella S., De Pastena M., D’Onofrio M., Crinò S.F., Pan T.L., De Robertis R., Elio G., Martone E., Bassi C., Salvia R. (2018). Palliative therapy in pancreatic cancer-interventional treatment with radiofrequency ablation/irreversible electroporation. Transl. Gastroenterol. Hepatol..

[33] Bagla S., Papadouris D. (2012). Percutaneous Irreversible Electroporation of Surgically Unresectable Pancreatic Cancer: A Case Report. J. Vasc. Interv. Radiol..

[34] Maor E., Ivorra A., Leor J., Rubinsky B. (2007). The Effect of Irreversible Electroporation on Blood Vessels. Technol. Cancer Res. Treat..

[35] Lafranceschina S., Brunetti O., Delvecchio A., Conticchio M., Ammendola M., Currò G., Piardi T., de’Angelis N., Silvestris N., Memeo R. (2019). Systematic Review of Irreversible Electroporation Role in Management of Locally Advanced Pancreatic Cancer. Cancers.

[36] Klaiber U., Schnaidt E., Hinz U., Gaida M., Heger U., Hank T., Strobel O., Neoptolemos J.P., Mihaljevic A.L., Büchler M.W. (2019). Prognostic Factors of Survival After Neoadjuvant Treatment and Resection for Initially Unresectable Pancreatic Cancer. Ann. Surg..

[37] Vogel J.A., Rombouts S.J., de Rooij T., van Delden O.M., Dijkgraaf M.G., van Gulik T.M., van Hooft J.E., van Laarhoven H.W., Martin R.C., Schoorlemmer A. (2017). Induction Chemotherapy Followed by Resection or Irreversible Electroporation in Locally Advanced Pancreatic Cancer (IMPALA): A Prospective Cohort Study. Ann. Surg. Oncol..

[38] Ratnayake B., Savastyuk A.Y., Nayar M., Wilson C.H., Windsor J.A., Roberts K., French J.J., Pandanaboyana S. (2020). Recurrence Patterns for Pancreatic Ductal Adenocarcinoma after Upfront Resection Versus Resection Following Neoadjuvant Therapy: A Comprehensive Meta-Analysis. J. Clin. Med..

[39] Scheffer H.J., Stam A.G.M., Geboers B., Vroomen L.G.P.H., Ruarus A., de Bruijn B., van den Tol M.P., Kazemier G., Meijerink M.R., de Gruijl T.D. (2019). Irreversible electroporation of locally advanced pancreatic cancer transiently alleviates immune suppression and creates a window for antitumor T cell activation. Oncoimmunology.

[40] Narayanan J.S.S., Ray P., Hayashi T., Whisenant T.C., Vicente D., Carson D.A., Miller A.M., Schoenberger S.P., White R.R. (2019). Irreversible Electroporation Combined with Checkpoint Blockade and TLR7 Stimulation Induces Antitumor Immunity in a Murine Pancreatic Cancer Model. Cancer Immunol. Res..

[41] Izzo F., Granata V., Fusco R., D’Alessio V., Petrillo A., Lastoria S., Piccirillo M., Albino V., Belli A., Tafuto S. (2021). Clinical Phase I/II Study: Local Disease Control and Survival in Locally Advanced Pancreatic Cancer Treated with Electrochemotherapy. J. Clin. Med..

[42] Granata V., Fusco R., Setola S.V., Piccirillo M., Leongito M., Palaia R., Granata F., Lastoria S., Izzo F., Petrillo A. (2017). Early radiological assessment of locally advanced pancreatic cancer treated with electrochemotherapy. World J. Gastroenterol. WJG.

[43] Casadei R., Ricci C., Ingaldi C., Alberici L., Di Marco M., Guido A., Minni F., Serra C. (2020). Intraoperative electrochemotherapy in locally advanced pancreatic cancer: Indications, techniques and results-a single-center experience. Updates Surg..

