# Supplementary material for: Margin Accentuation Irreversible Electroporation in Stage III Pancreatic Cancer: A Systematic Review
Source: Cancers (Basel). 2021 Jun 27;13(13):3212. doi: 10.3390/cancers13133212 (PMC8268790; doi:10.3390/cancers13133212)
Supplement: Supplementary file 1 [file cancers-13-03212-s001.zip › cancers-1240606-supplementary.pdf]

cancers

MDPI

Supplementary Materials: Margin Accentuation Irreversible Electroporation in Stage III Pancreatic Cancer: A Systematic Review

Bathiya Ratnayake, Dhya Al-Leswas, Ghazaleh Mohammadi-Zaniani, Peter Littler, Gourab Sen, Derek Manas and Sanjay Pandanaboyana

Table S1. Methodological items for non- randomized studies criteria (MINORS) scores among all domains for all included studies.

Methodological items for non- randomized studies	Martin et al (2012) [1]	Martin et al (2013) [2]	Kwon et al (2014) [3]	Dunki-Jacobs et al (2014) [4]	Martin et al (2015) [5]	Kluger et al (2016) [6]	Marsanic et al (2017) [7]	Kluger et al (2018) [8]
A clearly stated aim	2	1	2	2	1	2	1	2
Inclusion of consecutive patients	2	2	2	2	1	2	1	2
Prospective collection of data	2	1	1	1	1	1	1	2
Endpoints appropriate to the aim of the study	2	2	2	2	1	2	1	2
Unbiased assessment of the study endpoint	1	1	2	2	0	1	0	2
Follow-up period appropriate to the aim of the study	2	1	2	2	1	1	1	1
Loss to follow up less than 5%	2	1	2	2	2	2	2	2
Prospective calculation of the study size	0	0	0	0	0	0	0	0
Additional criteria for comparative study								
An adequate control group	-	0	-	-	0	0	-	-
Contemporary groups	-	0	-	-	0	0	-	-
Baseline equivalence of groups	-	0	-	-	0	0	-	-
Adequate statistical analyses	-	0	-	-	0	0	-	-
Total	11	9	13	13	7	11	7	13

Reference

1. Martin, R.C.; McFarland, K.; Ellis, S.; Velanovich, V. Irreversible Electroporation Therapy in the Management of Locally Advanced Pancreatic Adenocarcinoma. *J. Am. Coll. Surg.* **2012**, *215*, 361–369, doi:10.1016/j.jamcollsurg.2012.05.021.
2. Martin, R.C.G.; McFarland, K.; Ellis, S.; Velanovich, V. Irreversible Electroporation in Locally Advanced Pancreatic Cancer: Potential Improved Overall Survival. *Ann. Surg. Oncol.* **2012**, *20*, 443–449, doi:10.1245/s10434-012-2736-1.
3. Kwon, D.; McFarland, K.; Velanovich, V.; Martin, R.C. Borderline and locally advanced pancreatic adenocarcinoma margin accentuation with intraoperative irreversible electroporation. *Surg.* **2014**, *156*, 910–922, doi:10.1016/j.surg.2014.06.058.
4. Dunki-Jacobs, E.M.; Philips, P.; Martin, R.C. Evaluation of Resistance as a Measure of Successful Tumor Ablation During Irreversible Electroporation of the Pancreas. *J. Am. Coll. Surg.* **2014**, *218*, 179–187, doi:10.1016/j.jamcollsurg.2013.10.013.
5. Martin, R.C.G.; Kwon, D.; Chalikonda, S.; Sellers, M.; Kotz, E.; Scoggins, C.; McMasters, K.M.; Watkins, K. Treatment of 200 Locally Advanced (Stage III) Pancreatic Adenocarcinoma Patients With Irreversible Electroporation. *Ann. Surg.* **2015**, *262*, 486–494, doi:10.1097/sla.0000000000001441.
6. Kluger, M.D.; Epelboym, I.; Schrope, B.A.; Mahendraraj, K.; Hecht, E.M.; Susman, J.; Weintraub, J.L.; Chabot, J.A. Single-Institution Experience with Irreversible Electroporation for T4 Pancreatic Cancer: First 50 Patients. *Ann. Surg. Oncol.* **2015**, *23*, 1736–1743, doi:10.1245/s10434-015-5034-x.
7. Marsanic, P.; Mellano, A.; Sottile, A.; De Simone, M. Irreversible electroporation as treatment of locally advanced and as margin accentuation in borderline resectable pancreatic adenocarcinoma. *Med Biol. Eng. Comput.* **2017**, *55*, 1123–1127, doi:10.1007/s11517-016-1603-9.
8. Kluger, M.D.; Rashid, M.F.; Rosario, V.L.; Schrope, B.A.; Steinman, J.A.; Hecht, E.M.; Chabot, J.A. Resection of Locally Advanced Pancreatic Cancer without Regression of Arterial Encasement After Modern-Era Neoadjuvant Therapy. *J. Gastrointest. Surg.* **2018**, *22*, 235–241, doi:10.1007/s11605-017-3556-1.
